# Growth mindset and student writing feedback literacy among Chinese learners of Japanese: the mediating role of achievement goals

**DOI:** 10.3389/fpsyg.2026.1758874

**Published:** 2026-02-02

**Authors:** Xi Chen, Xue Bai, Lu Li, Xinying Liu

**Affiliations:** 1 School of Japanese Culture and Economics, Xi’an International Studies University, Xi’an, Shaanxi, China; 2 Graduate School, Xi’an International Studies University, Xi’an, Shaanxi, China; 3 School of Foreign Languages, Shanghai University, Shanghai, China

**Keywords:** achievement goals, growth mindset, Japanese learners in China, L2 writing, student writing feedback literacy

## Abstract

**Introduction:**

Research on student writing feedback literacy is increasing, but individual learner factors remain underexamined, and most existing studies focus on English learners rather than Japanese learners. This study explores the relationships between Japanese learners’ growth mindset and student writing feedback literacy, as well as the mediating role of achievement goals.

**Methods:**

Data were collected through a questionnaire completed by 309 Japanese learners in China. The survey assessed growth mindset, achievement goals, and student writing feedback literacy. Structural equation modeling (SEM) and bootstrapped mediation analysis were used to examine the direct and indirect relationships among the variables.

**Results:**

SEM results revealed that the direct effect of growth mindset on student writing feedback literacy was not significant. Mediation analyses showed that development-approach, development-avoidance, and demonstration-approach goals positively mediated the relationship between growth mindset and student writing feedback literacy, with all indirect effects being statistically significant.

**Discussion:**

Achievement goals serve as a key mediating mechanism linking Japanese learners’ growth mindset and student writing feedback literacy. Such practices as development-oriented instructional goals and formative assessment may provide a supportive context for fostering a growth mindset and development-focused achievement goals, thereby supporting the development of student writing feedback literacy.

## 1 Introduction

Feedback in second language (L2) writing has been conceptualized as a constructive judgment of written texts that guides students toward improving their future writing and developing their writing processes (Hyland and Hyland, 2019). Given its crucial role, writing feedback has long been a central concern of L2 writing research (Yu et al., 2023; Zhang and Mao, 2023). Earlier research on writing feedback has primarily focused on improving the quality of feedback (Liu and Yu, 2022), including types of feedback (Ellis, 2009), foci (Bitchener and Knoch, 2010), and feedback providers (Lam, 2013), but has paid relatively little attention to students’ roles in understanding and using feedback (Li and Vuono, 2019). With the shift in higher education from a one-way transmissive model of feedback to a learner-centered dialogic model (Boud and Molloy, 2013; Carless and Boud, 2018), increasing attention has been directed to student writing feedback literacy, which is defined as L2 students’ knowledge, beliefs, practices, abilities, and skills involved in appreciating, evaluating, and using L2 writing feedback, as well as in managing the emotions in this process (Yu and Liu, 2021; Yu et al., 2022). Student writing feedback literacy contributes to feedback effectiveness by helping students appreciate feedback and use it as a resource for improving their writing, thereby bridging the gap between their current performance and desired achievement (Yu and Liu, 2021; Yu et al., 2022; Boggs and Manchón, 2023; Zhang et al., 2024).

Students’ beliefs and goals are key individual factors that shape how they appreciate, evaluate, and act on writing feedback. Specifically, these factors influence whether students perceive feedback as a learning opportunity, as well as their cognitive, emotional, and behavioral engagement with feedback, and the extent of its uptake and retention (Storch and Wigglesworth, 2010; Han and Hyland, 2015; Han, 2017, 2019). In terms of beliefs, one core component is students’ mindsets, which are relatively stable meaning systems that center on the question of whether students’ abilities can be changed (Dweck, 2006). Students with a fixed mindset view language ability as innate and unchangeable, whereas those with a growth mindset believe it is malleable and can be improved through effort and effective strategies (Lou and Noels, 2017, 2019). Research has shown that a growth mindset has positive effects on students’ perceptions of feedback value (Papi et al., 2020; Yao et al., 2020), their feedback-seeking behaviors (Waller and Papi, 2017; Xu, 2022; Xu and Wang, 2023), and their student writing feedback literacy in L1 writing contexts (Zhu et al., 2024). In comparison, achievement goals reflect students’ motivational orientations in specific learning situations, specifying what they strive for when they engage in a task (Elliot and Church, 1997). Within achievement goal theory, development goals emphasize improving competence, whereas demonstration goals focus on demonstrating competence (Korn and Elliot, 2016). According to the social-cognitive model, students’ mindsets shape how they interpret effort, failure, and evaluation in achievement contexts, thereby guiding the adoption of different types of achievement goals in specific situations. These goals, in turn, influence students’ cognitive, emotional, and behavioral responses when they encounter challenging tasks (Dweck and Leggett, 1988). Research has shown that achievement goals mediate the relationship between mindsets and writing performance (Limpo and Alves, 2017; Camacho et al., 2022), self-regulated writing strategies (Zhang and Jiang, 2024), and feedback-seeking behavior (Sun and Huang, 2023; Yao and Zhu, 2024). These findings suggest that achievement goals serve as a key motivational mechanism linking mindsets to students’ cognitive, emotional, and behavioral responses in writing-related contexts.

Due to the dominance of English in global language education, existing L2 writing research has primarily focused on English learners, while paying insufficient attention to learners of languages other than English (LOTE). However, LOTE learners face distinct challenges, as they write in diverse contexts and in languages that are not globally dominant (Reichelt, 2016; Yiğitoğlu and Reichelt, 2019). These challenges underscore the limitations of English-based research, which, despite providing valuable insights, cannot fully account for the characteristics of LOTE learners (Chen et al., 2020; Bruton et al., 2010; Hedgcock and Lefkowitz, 1994, 1996). For example, compared to English learners, LOTE learners, such as those studying French and Spanish, often believe that their target languages have limited utility for future career development (Chen et al., 2020). Spanish learners are less likely than English learners to recognize the actual value of writing. They are more inclined to question it, as they expect fewer opportunities to use it in their future careers (Bruton et al., 2010). In terms of writing feedback, LOTE learners (French, Spanish, and German) show a stronger preference for written feedback, relying more on explicit lexical and grammatical corrections and tending to view writing as a form-focused practice. In contrast, English learners place greater emphasis on discourse organization and clarity of expression, prefer more comprehensive feedback, and often view writing as a means of expressing ideas and improving overall writing ability (Hedgcock and Lefkowitz, 1994, 1996). These differences highlight the importance of examining writing feedback among LOTE learners, thereby contributing to a more comprehensive understanding of L2 writing (Reichelt, 1999) and responding to calls for broadening the scope of L2 writing research (Manchón, 2012).

According to The Japan Foundation (2021), the number of Japanese learners in China is the highest worldwide, surpassing 1.05 million, with more than half studying Japanese at higher education institutions. Japanese has become the second-most widely learned foreign language in China, after English. Unlike English, which is typically taught at earlier stages of education, Japanese is generally introduced at the university stage, starting from the beginner level (Li et al., 2026). For Japanese learners at this stage, writing constitutes a highly challenging task, as it imposes substantial cognitive demands and readily exposes errors. Accordingly, it is necessary to attend to student writing feedback literacy, as it can help learners interpret, utilize, and respond to feedback more effectively, thereby supporting the development of their writing ability at this critical stage. The present study focuses on Japanese learners and explores individual factors associated with student writing feedback literacy, with particular attention to growth mindset and achievement goals.

## 2 Literature review

### 2.1 Student writing feedback literacy

Feedback literacy was first proposed by Sutton (2012), who defined it as “the ability to read, interpret, and use written feedback.” Carless and Boud (2018) extended this concept to the field of education by introducing the notion of student feedback literacy, conceptualized as the understandings, capacities, and dispositions necessary for interpreting feedback and using it to enhance academic work and learning strategies. Based on this definition, they outlined four interrelated features that form a conceptual framework for student feedback literacy: appreciating feedback, making judgments, managing affect, and taking action. These studies provide a foundation for examining student feedback literacy in L2 writing contexts. Subsequently, researchers have further explored the characteristics of student feedback literacy in L2 writing (Han and Xu, 2021; Li and Han, 2022; Yu et al., 2022). Drawing on case-study evidence, Han and Xu (2021) identified cognitive capacity, socio-affective disposition, and socio-affective capacity as key components of student writing feedback literacy, whereas Li and Han (2022) outlined a two-dimensional structure consisting of cognitive readiness and socio-affective readiness. Both studies also emphasized that individual factors, such as subject knowledge (Li and Han, 2022) and learner beliefs (Han and Xu, 2021), play an essential role in shaping the development of student writing feedback literacy. In contrast to case-study approaches, Yu et al. (2022) developed and validated a large-scale questionnaire based on Carless and Boud (2018) framework. Their scale expanded student writing feedback literacy into five dimensions: appreciating feedback (i.e., how students perceive the value of feedback and their roles in the feedback process), acknowledging different feedback sources (i.e., how students understand the learning benefits offered by different sources of feedback), making judgments (i.e., how students evaluate feedback and decide what to use), managing affect (i.e., how students regulate their emotions during the feedback process), and taking action (i.e., how students act on the feedback they have received). The scale provides a reliable tool for subsequent quantitative investigations. Overall, existing research indicates that student writing feedback literacy is a multidimensional construct encompassing cognitive, affective, and behavioral aspects, and that individual factors play a significant role in its development.

Recently, a few studies have sought to examine how individual factors influence student writing feedback literacy, focusing on two types of variables: cognitive factors (Zhang et al., 2024; Zhou et al., 2022) and socio-affective factors (Xu and Feng, 2025; Zhu et al., 2024). Regarding cognitive factors, Zhang et al. (2024) found that language proficiency has a significant impact on the development of student writing feedback literacy. Low-proficiency students showed limited improvement. Middle-proficiency students improved in appreciating feedback, acknowledging different feedback sources, managing affect, and taking action, whereas high-proficiency students improved only in appreciating feedback. Similarly, Zhou et al. (2022) reported that students with stronger writing ability demonstrated more advanced student writing feedback literacy, characterized by proactive engagement with feedback, clearer evaluative standards, and more extensive revisions, whereas students with weaker writing ability, despite recognizing the value of feedback, were less prepared to benefit from it. With respect to socio-affective factors, Xu and Feng (2025) found that both ideal L2 writing self and writing persistence positively predicted student writing feedback literacy. Their mediation analysis further revealed that writing persistence mediated the effects of both ideal L2 writing self and ought-to L2 writing self on student writing feedback literacy. Zhu et al. (2024) found that students’ growth mindset predicted all five dimensions of student writing feedback literacy and indirectly enhanced feedback engagement, indicating that growth mindset is a crucial socio-affective factor. However, since Zhu et al. (2024) conducted their study in an L1 writing context, it is still uncertain whether the effects of a growth mindset on student writing feedback literacy can be generalized to L2 writing contexts.

### 2.2 Growth mindset and student writing feedback literacy

A growth mindset enables students to view L2 learning as an opportunity for competence development, embrace challenges, and learn from failure (Lou and Noels, 2019). Existing L2 writing studies have demonstrated that a growth mindset is associated with multiple dimensions of student writing feedback literacy proposed by Yu et al. (2022). To begin with, a growth mindset enhances students’ ability to appreciate feedback and make judgments. Students with a growth mindset are more likely to recognize the value of feedback and view it as an opportunity for improvement, leading them to engage more deeply with evaluative information (Papi et al., 2020; Yao et al., 2020). Furthermore, students with a growth mindset demonstrate stronger competence in acknowledging different feedback sources. They tend to show more positive attitudes toward feedback from diverse providers, including teacher and peer feedback, and are therefore more willing to learn from multiple perspectives (Papi et al., 2020; Yao et al., 2020; Hwang, 2025). In addition, a growth mindset contributes to students’ ability to manage affect. Hwang (2025) observed that students with a growth mindset experience more positive emotions and possess stronger emotion-regulation skills, which help them cope with anxiety, frustration, and other negative emotions during the writing process. Wang (2025) similarly found that the more students believe in the malleability of their writing competence, the more enjoyment they report in L2 writing classes. Finally, a growth mindset promotes students’ capacity to take action on feedback. Students with a growth mindset tend to actively use feedback, seeking it through feedback monitoring and inquiry as part of their efforts to improve their writing (Waller and Papi, 2017; Xu, 2022; Xu and Wang, 2023). While the above studies demonstrate that a growth mindset has positive effects on students’ cognitive, emotional, and behavioral responses in the L2 writing feedback process, the existing evidence is dispersed across separate studies, and its overall influence on student writing feedback literacy has yet to be clearly established.

### 2.3 Achievement goals and student writing feedback literacy

Achievement goal theory has undergone substantial refinement over time (Elliot and Hulleman, 2017). Achievement goals refer to individuals’ purposes for engaging in achievement-relevant behaviors and were initially classified into mastery goals and performance goals (Dweck, 1986; Dweck and Leggett, 1988; Ames, 1992). Building on this distinction, Elliot and Harackiewicz (1996) introduced the approach-avoidance dimension and further divided performance goals into performance-approach and performance-avoidance goals, forming the trichotomous achievement goal model. Later, Korn and Elliot (2016) relabeled mastery and performance goals as development goals and demonstration goals, based on the core characteristics of early achievement goals, and applied the approach–avoidance distinction to both types of goals, thereby proposing a 2 × 2 achievement goal model. Within this model, development-approach goals focus on improving competence, whereas development-avoidance goals emphasize preventing decline; demonstration-approach goals involve showing competence to gain positive evaluations, while demonstration-avoidance goals involve avoiding negative assessments. Following this model, the present study adopts the development/demonstration terminology for achievement goals. Research based on the 2 × 2 model indicates that learners with different goal orientations exhibit distinct motivational and performance patterns when facing academic challenges (Korn and Elliot, 2016; Elliot and Hulleman, 2017). Specifically, development-approach goals are consistently associated with higher intrinsic motivation, though their effects on academic performance are less robust. Development-avoidance goals may also be linked to intrinsic motivation but tend to show limited or inconsistent effects on performance. In contrast, demonstration-approach goals are often unrelated to intrinsic motivation but can facilitate short-term performance outcomes, whereas demonstration-avoidance goals are generally the most maladaptive, undermining both motivation and achievement (Korn and Elliot, 2016).

In L2 feedback contexts, the 2 × 2 achievement goal model provides a key framework for explaining students’ behavioral responses (Papi et al., 2019; Sun and Huang, 2023; Papi et al., 2021). Papi et al. (2019) were the first to apply this model in L2 settings. Their findings showed that students with development-approach goals tended to engage in both feedback monitoring and feedback inquiry. By contrast, students with demonstration-approach goals were more inclined to seek feedback from teachers, while those with demonstration-avoidance goals preferred to obtain information from peers. Development-avoidance goals did not predict any form of feedback-seeking behavior. Sun and Huang (2023) further validated this model among Chinese EFL students, showing that development-approach goals significantly predicted feedback monitoring, whereas demonstration-approach goals significantly predicted both direct and indirect inquiry. Neither avoidance-oriented goal predicted feedback-seeking behavior. Notably, both studies demonstrated that development-approach and demonstration-approach goals partially mediated the relationship between growth mindset and feedback-seeking behavior, underscoring the central motivational role of achievement goals in this process. Moreover, Papi et al. (2021) found that different achievement goals also differentiated students’ feedback preferences: students with development-approach goals preferred explicit corrective feedback, those with development-avoidance goals preferred implicit corrective feedback, demonstration-approach goals did not significantly predict any feedback type, and students with demonstration-avoidance goals were more likely to avoid corrective feedback. Although existing research consistently suggests that development-approach goals tend to be the most adaptive and demonstration-avoidance goals the most maladaptive, the effects of development-avoidance goals appear more complex and unstable across studies. For example, development-avoidance goals may be positively associated with intrinsic motivation in some contexts but play only a limited role when L2 students deal with feedback, indicating a strong context-dependent nature. Therefore, how the four types of achievement goals shape students’ understanding, evaluation, and use of feedback in the more complex context of L2 writing feedback remains to be further examined.

### 2.4 Growth mindset, achievement goals, and student writing feedback literacy

Mindsets are an important antecedent to achievement goals. Students’ mindsets shape their achievement goal orientations, which, in turn, influence their cognitive, emotional, and behavioral responses to challenging tasks (Dweck and Leggett, 1988). On the one hand, students with a growth mindset hold a general belief that ability can be continuously developed through effort; they are more likely to construe achievement contexts as opportunities for competence development and information acquisition. Therefore, in specific contexts, they set development goals aimed at improving competence, which provide a directional orientation for regulating their learning activities. Such students tend to focus on developing their competence, regulating their emotions effectively, maintaining positive learning experiences, and engaging persistently in learning activities (Zeng et al., 2016; Lou and Noels, 2017, 2019; Ma et al., 2024). They also demonstrate better writing performance (Limpo and Alves, 2017; Camacho et al., 2022). On the other hand, students with a fixed mindset view ability as relatively stable and difficult to change, and tend to interpret achievement situations as contexts for testing or evaluating their ability level, placing greater emphasis on evaluative outcomes. Early research suggested that students with a fixed mindset were more likely to adopt demonstration goals centered on demonstrating or protecting their perceived competence, and that such goals could give rise to maladaptive outcomes, including avoidance of challenges, negative emotional experiences, and reduced engagement (Dweck and Leggett, 1988; Dweck, 1999). However, as noted by Dweck and Leggett (1988), demonstration-oriented goals are not inherently maladaptive. Evaluating one’s own ability and obtaining positive recognition of one’s competence from others are also necessary in many achievement contexts. This argument applies specifically to demonstration-approach goals, which are oriented toward attaining positive evaluations. Research has shown that students with a growth mindset may also pursue demonstration-approach goals (Molden and Dweck, 2000; Yu and McLellan, 2020). A key reason is that a growth mindset reshapes the psychological meaning of demonstration-approach goals and their achievement-related consequences (Molden and Dweck, 2000). Students with a growth mindset tend to interpret achievement situations as opportunities to obtain information about their current level of competence. Demonstration-approach goals no longer signify a final demonstration of fixed ability, but involve displaying one’s current level of task performance, understood as the product of sustained effort and remaining open to further development (Yu and McLellan, 2020). Failure to attain such goals is not necessarily detrimental; instead, it may provide students with diagnostic information about their academic skill levels (Molden and Dweck, 2000). Accordingly, students with a growth mindset may pursue both development goals and demonstration-approach goals, which can jointly support adaptive cognitive, emotional, and behavioral responses in achievement contexts.

Research on L2 writing also supports this perspective (Zhang and Jiang, 2024; Yao and Zhu, 2024). L2 students with a growth mindset tend to adopt development goals and demonstration-approach goals, which have been shown to mediate the relationship between growth mindset and self-regulated writing strategies (Zhang and Jiang, 2024) as well as feedback-seeking behavior (Yao and Zhu, 2024).

Collectively, these studies provide evidence for understanding the mediating role of achievement goals in linking growth mindset with processes of L2 writing feedback. Since existing studies were grounded in the trichotomous model, they paid limited attention to the potential role of development-avoidance goals in writing feedback. Given that development-avoidance goals have shown complex and unstable effects across different learning contexts, their influence in L2 writing feedback has not yet been fully clarified. To develop a more comprehensive understanding of how a growth mindset shapes students’ cognitive, affective, and behavioral responses during writing feedback through different goal pathways, it is essential to adopt the 2 × 2 achievement goal model and examine the potential mediating roles of all four types of achievement goals in the relationship between growth mindset and student writing feedback literacy.

### 2.5 The present study

**Figure 1:**
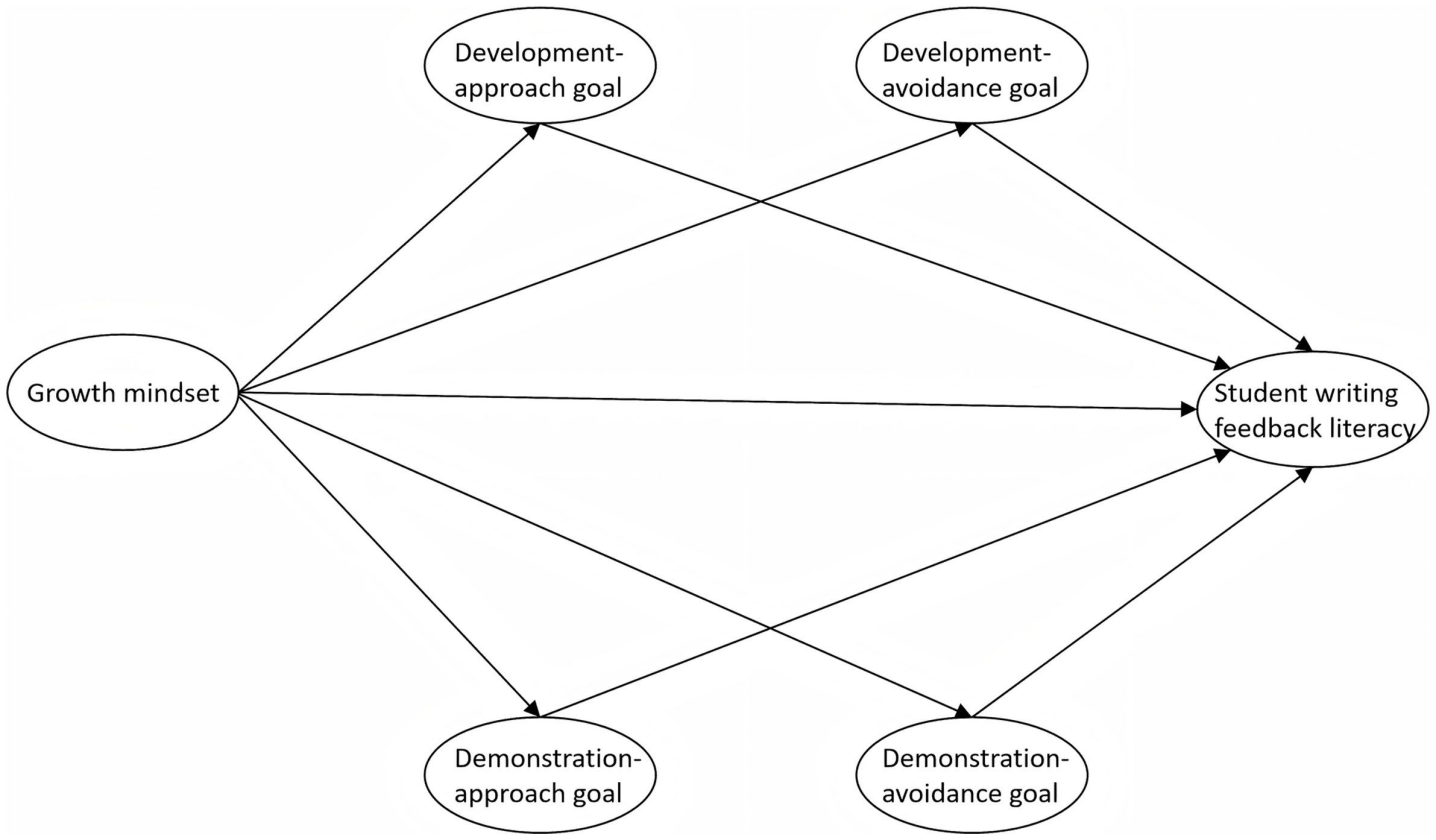
Hypothesized conceptual framework of growth mindset, achievement goals, and student writing feedback literacy.

Despite growing evidence supporting the positive role of a growth mindset in L2 writing feedback contexts, its influence on student writing feedback literacy remains unclear, particularly with respect to the potential mediating role of achievement goals. Moreover, current research has focused on English learners or general L2 learning contexts, with relatively limited attention to the writing feedback processes of LOTE learners, such as Japanese learners. Against this backdrop, the present study focuses on Japanese learners, drawing on the 2 × 2 achievement goal model, proposes a hypothesized conceptual framework that examines the relationship between growth mindset and student writing feedback literacy through both direct and indirect pathways, with achievement goals serving as key mediators (see Figure 1). The specific research questions are as follows:

*RQ1*: Is a growth mindset directly associated with student writing feedback literacy?

*RQ2*: Is the relationship between growth mindset and student writing feedback literacy mediated by achievement goals?

## 3 Methods

### 3.1 Participants

This study employed convenience sampling, recruiting students majoring in Japanese from a university in Xi’an, Shaanxi Province, China. A total of 309 valid responses were collected through Wenjuanxing, an online survey platform widely used in China.^1^ The participants, aged 20 to 29 years (M = 22.097, SD = 2.057), included 76 males (25%) and 233 females (75%). They were third- or fourth-year undergraduates and graduate students with comparable learning backgrounds, having studied Japanese for an average of 3.981 years (SD = 1.319), and all had completed at least one Japanese writing course.

Ethical approval for this study was obtained through the ethics review process of the authors’ affiliated school. The survey was administered during class sessions. Before completing the questionnaire, participants were informed of the study’s purpose and the intended use of their data. Standardized instructions were provided to ensure comprehension of all questionnaire items.

### 3.2 Instruments

The study developed a composite questionnaire based on established instruments, consisting of four sections: demographic information, growth mindset, achievement goals, and student writing feedback literacy. All questionnaire items were presented in Chinese to facilitate comprehension. A back-translation procedure was employed. Specifically, two authors independently translated the original English scales into Chinese and resolved discrepancies through discussion. A doctoral student in foreign language education then back-translated the consolidated Chinese version of the scales into English. The research team compared the back-translated version with the original scales and revised the Chinese version to minimize semantic deviations. Furthermore, considering that the participants were Japanese language learners, phrases such as “learning Japanese” and “Japanese writing” were incorporated into some items to enhance contextual relevance.

Growth mindset was measured using the “Second Language Aptitude Beliefs (L2B)” subscale developed by Lou and Noels (2017). The growth mindset dimension comprises 3 items, each rated on a 6-point Likert scale ranging from 1 (strongly disagree) to 6 (strongly agree). The present study focused on these 3 items to align with the research aims and minimize participant burden in a questionnaire that included multiple constructs. A sample item is “You can always change your foreign language ability”. In the present study, Cronbach’s alpha was 0.793, suggesting acceptable internal consistency.

Achievement goals were assessed using the “Achievement Goal Questionnaire” developed by Korn and Elliot (2016) and adapted to the context of Japanese language learning. The scale comprised 12 items across four dimensions: Development-approach goals (e.g., “My focus is to develop my Japanese knowledge”), Development-avoidance goals (e.g., “My goal is to avoid a decrease in my Japanese ability”), Demonstration-approach goals (e.g., “My focus is to demonstrate that I am knowledgeable in Japanese”), and Demonstration-avoidance goals (e.g., “My aim is to avoid showing incompetence in Japanese”). Responses were rated on a 5-point Likert scale ranging from 1 (strongly disagree) to 5 (strongly agree). In the present study, Cronbach’s alpha for the four subscales was 0.797, 0.847, 0.760, and 0.894, indicating good internal consistency.

Student writing feedback literacy was assessed using the “L2 Student Writing Feedback Literacy Scale” developed by Yu et al. (2022) and adapted to the context of Japanese language learning. Following exploratory factor analysis, 24 items were retained across five dimensions: Appreciating Feedback (e.g., “I think that feedback can improve my Japanese writing skills”), Acknowledging Different Feedback Sources (e.g., “I think that technology can be used to access, store, and revisit feedback”), Making Judgments (e.g., “I appreciate the role of feedback standards and criteria in judging the work of others”), Managing Affect (e.g., “I can maintain emotional equilibrium and avoid defensiveness when receiving critical feedback”), and Taking Action (e.g., “I can draw inferences from a range of Japanese writing feedback experiences for the purpose of continuous improvement”). Responses were rated on a 5-point Likert scale ranging from 1 (strongly disagree) to 5 (strongly agree). In the present study, Cronbach’s alpha for the five subscales was 0.899, 0.834, 0.820, 0.783, and 0.864, suggesting acceptable internal consistency.

### 3.3 Data analysis

Descriptive statistics and correlational analyses were first conducted using SPSS 27.0. Following the two-step approach proposed by Anderson and Gerbing (1988), the measurement and structural models were tested separately in AMOS 24.0. To ensure the construct validity of the measurement model, confirmatory factor analysis (CFA) was performed, and convergent and discriminant validity were evaluated based on factor loadings, composite reliability (CR), and average variance extracted (AVE), respectively. Subsequently, the structural model was examined to assess the relationships among the latent variables. The model fit was evaluated using multiple indices, including χ^2^/df < 3 (Kline, 2016), CFI and TLI ≥ 0.95, RMSEA ≤ 0.06, and SRMR ≤ 0.08 (Hu and Bentler, 1999). Finally, the mediating effects were tested using the bootstrap method (5,000 resamples, 95% confidence intervals).

## 4 Results

### 4.1 Common method bias test

All data in this study were collected through self-reported questionnaires; therefore, Harman’s single-factor test was used to assess common method bias. The results showed that the first factor accounted for 31% of the variance, which was below the 40% critical threshold, indicating that common method bias was not a major concern in this study.

### 4.2 Descriptive statistics and correlation analysis

Table 1 shows the means, standard deviations, and correlations of the main variables. The absolute values of skewness and kurtosis for all variables were below 2, indicating a normal data distribution. Descriptive statistics indicated mean scores of 3.964 (SD = 0.795) for growth mindset and 3.839 (SD = 0.540) for student writing feedback literacy. Among the achievement goals, development-approach goals showed the highest mean score (M = 4.121, SD = 0.578), followed by demonstration-approach goals (M = 3.899, SD = 0.644) and development-avoidance goals (M = 3.419, SD = 0.868). In contrast, demonstration-avoidance goals had the lowest mean score (M = 3.084, SD = 1.003).

**Table 1:** Descriptive statistics and correlation.

Variable	M	SD	1	2	3	4	5	6
1. Growth mindset	3.964	0.795	1					
2. Development-approach goal	4.121	0.578	0.278^**^	1				
3. Development-avoidance goal	3.419	0.868	0.152^**^	0.198^**^	1			
4. Demonstration-approach goal	3.899	0.644	0.452^**^	0.404^**^	0.202^**^	1		
5. Demonstration-avoidance goal	3.084	1.003	0.027	0.101	0.314^**^	0.065	1	
6. Student writing feedback literacy	3.839	0.540	0.364^**^	0.497^**^	0.254^**^	0.494^**^	0.010	1

**p < 0.01.

Correlation analysis results showed that growth mindset was positively correlated with development-approach goals (*r* = 0.278, *p* < 0.01), development-avoidance goals (*r* = 0.152, *p* < 0.01), demonstration-approach goals (*r* = 0.452, *p* < 0.01), and student writing feedback literacy (*r* = 0.364, *p* < 0.01). Student writing feedback literacy was positively correlated with development-approach goals (*r* = 0.497, *p* < 0.01), development-avoidance goals (*r* = 0.254, *p* < 0.01), and demonstration-approach goals (*r* = 0.494, *p* < 0.01).

### 4.3 Structural model analysis

Before testing the structural model, confirmatory factor analysis (CFA) was conducted to examine the measurement model, which comprised six latent variables: growth mindset, development-approach goals, development-avoidance goals, demonstration-approach goals, demonstration-avoidance goals, and student writing feedback literacy. The results indicated a satisfactory model fit: χ^2^/df = 1.230, CFI = 0.987, TLI = 0.984, RMSEA = 0.027, SRMR = 0.040. All factor loadings of the observed variables were statistically significant (*p* < 0.001). As shown in Table 2, the factor loadings ranged from 0.638 to 0.884, all exceeding the recommended threshold of 0.50 (Hair et al., 2019), indicating that the observed variables effectively represented their corresponding latent constructs. In addition, the composite reliability (CR) values of the latent constructs ranged from 0.766 to 0.895, exceeding the threshold of 0.70, while the average variance extracted (AVE) values ranged from 0.523 to 0.740, surpassing the recommended criterion of 0.50. These results demonstrate that the measurement model exhibited good internal consistency and satisfactory convergent validity (Hair et al., 2019). Discriminant validity was assessed by comparing the square root of AVE for each construct with the inter-construct correlations, following the criterion proposed by Fornell and Larcker (1981). As shown in Table 3, the square roots of the AVEs for all constructs were greater than the corresponding inter-construct correlations, demonstrating that each construct was distinct from the others. Therefore, the measurement model exhibited satisfactory discriminant validity.

**Table 2:** Convergent validity.

Construct	Factor loading (*λ*)	Composite reliability (CR)	Average variance extracted (AVE)
Growth mindset	[0.725–0.765]	0.794	0.563
Development-approach goal	[0.638–0.827]	0.801	0.576
Development-avoidance goal	[0.724–0.864]	0.849	0.652
Demonstration-approach goal	[0.662–0.786]	0.766	0.523
Demonstration-avoidance goal	[0.819–0.884]	0.895	0.740
Student writing feedback literacy	[0.646–0.832]	0.859	0.552

**Table 3:** Discriminant validity.

Construct	Square root of AVE	Correlation with other constructs
Growth mindset	0.750	0.353, 0.183, 0.575, 0.028, 0.443
Development-approach goal	0.759	0.353, 0.244, 0.492, 0.112, 0.598
Development-avoidance goal	0.808	0.183, 0.244, 0.258, 0.366, 0.279
Demonstration-approach goal	0.723	0.575, 0.492, 0.258, 0.081, 0.593
Demonstration-avoidance goal	0.860	0.028, 0.112, 0.366, 0.081, 0.016
Student writing feedback literacy	0.743	0.443, 0.598, 0.279, 0.593, 0.016

After confirming the satisfactory fit of the measurement model, structural equation modeling (SEM) was employed to investigate the relationships among growth mindset, achievement goals, and student writing feedback literacy.

**Figure 2:**
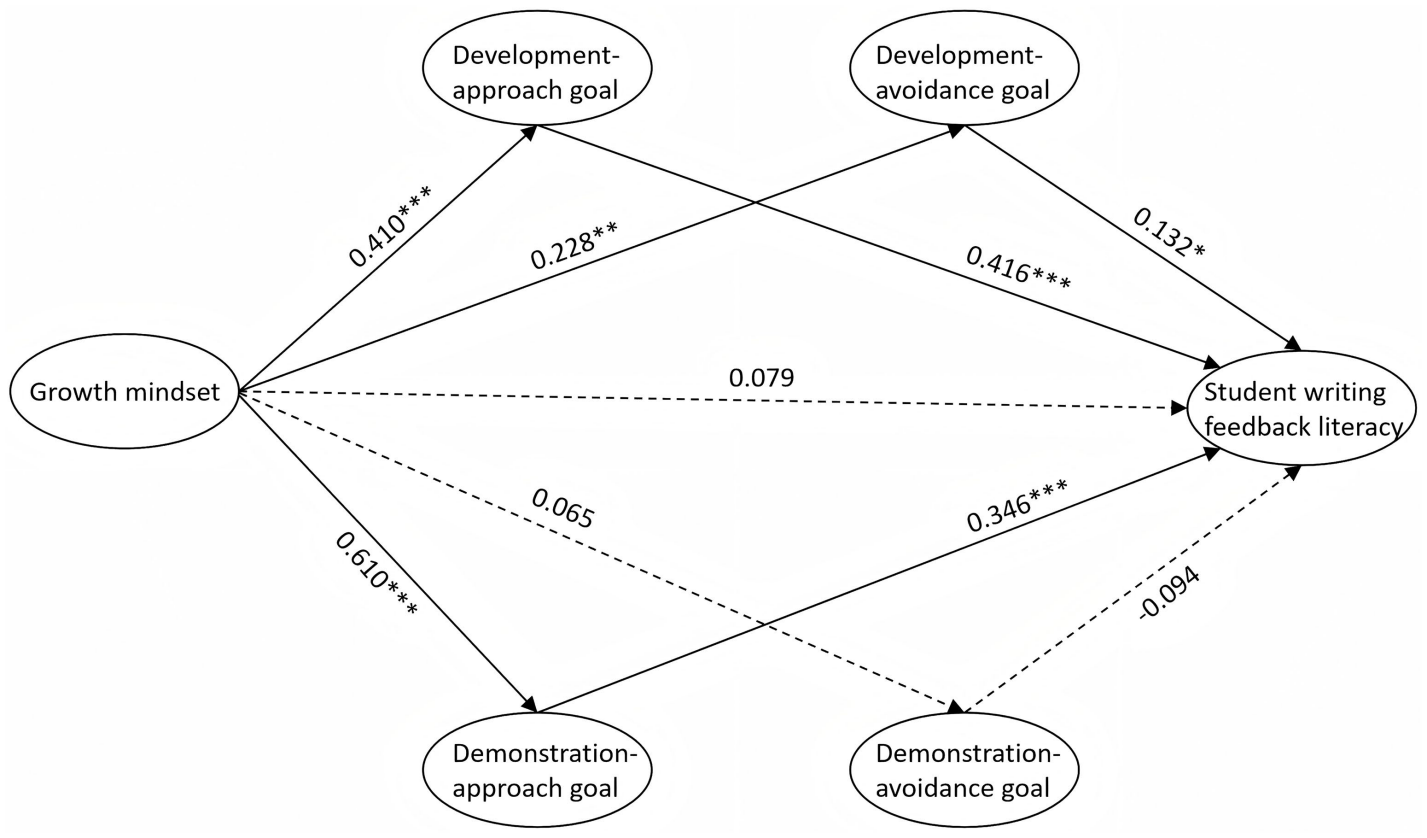
The structural model of growth mindset, achievement goals, and student writing feedback literacy.

The model tested both the direct effect of growth mindset on student writing feedback literacy and its indirect effects through the four types of achievement goals. These included development-approach, development-avoidance, demonstration-approach, and demonstration-avoidance goals, which were specified as parallel mediators. The structural model exhibited an acceptable fit to the data: χ^2^/df = 1.593, CFI = 0.965, TLI = 0.959, RMSEA = 0.044, SRMR = 0.080. As shown in Figure 2 and Table 4, growth mindset significantly predicted development-approach goals (*β* = 0.410, *p* < 0.001), development-avoidance goals (*β* = 0.228, *p* = 0.001), and demonstration-approach goals (*β* = 0.610, *p* < 0.001), whereas its effect on demonstration-avoidance goals was not significant (*β* = 0.065, *p* > 0.05). Development-approach goals (*β* = 0.416, *p* < 0.001), development-avoidance goals (*β* = 0.132, *p* < 0.05), and demonstration-approach goals (*β* = 0.346, *p* < 0.001) exerted significant positive effects on student writing feedback literacy, while demonstration-avoidance goals did not (*β* = −0.094, *p* > 0.05). The direct effect of growth mindset on student writing feedback literacy was not significant (*β* = 0.079, *p* > 0.05).

**Table 4:** Standardized path coefficients for the structural model.

Predictor	Outcome	*β*	SE	*p*
Growth mindset	Development-approach goal	0.410	0.052	< 0.001
Growth mindset	Development-avoidance goal	0.228	0.083	0.001
Growth mindset	Demonstration-approach goal	0.610	0.069	< 0.001
Growth mindset	Demonstration-avoidance goal	0.065	0.097	0.338
Development-approach goal	Student writing feedback literacy	0.416	0.092	< 0.001
Development-avoidance goal	Student writing feedback literacy	0.132	0.041	0.025
Demonstration-approach goal	Student writing feedback literacy	0.346	0.091	< 0.001
Demonstration-avoidance goal	Student writing feedback literacy	−0.094	0.032	0.087
Growth mindset	Student writing feedback literacy	0.079	0.068	0.340

To examine the mediating effects of achievement goals on the relationship between growth mindset and student writing feedback literacy, a bias-corrected bootstrap test (5,000 resamples) was conducted. Table 5 shows the results, which revealed that development-approach goals (*β* = 0.170, 95% CI [0.096, 0.283], *p* < 0.001), development-avoidance goals (*β* = 0.030, 95% CI [0.003, 0.080], *p* < 0.05), and demonstration-approach goals (*β* = 0.211, 95% CI [0.104, 0.373], *p* < 0.01) all exerted significant mediating effects, whereas the mediating effect of demonstration-avoidance goals was not significant (*β* = −0.006, 95% CI [−0.038, 0.005], *p* > 0.05). The direct effect of growth mindset on student writing feedback literacy was not significant, whereas the total indirect effect was significant (*β* = 0.406, *p* < 0.001), suggesting that the influence of growth mindset on student writing feedback literacy was fully mediated by achievement goals. The proportion of total effects indicated that the mediating effects were mainly derived from demonstration-approach goals (39%), development-approach goals (31%), and development-avoidance goals (6%).

**Table 5:** Indirect effects of growth mindset on student writing feedback literacy.

Indirect path	*β*	SE	95% BC CI	*p*
Growth mindset → Development-approach goal → Student writing feedback literacy	0.170	0.045	[0.096, 0.283]	< 0.001
Growth mindset → Development-avoidance goal → Student writing feedback literacy	0.030	0.019	[0.003, 0.080]	0.031
Growth mindset → Demonstration-approach goal → Student writing feedback literacy	0.211	0.068	[0.104, 0.373]	0.001
Growth mindset → Demonstration-avoidance goal → Student writing feedback literacy	−0.006	0.010	[−0.038, 0.005]	0.257

## 5 Discussion

### 5.1 Direct relationships among growth mindset, achievement goals, and student writing feedback literacy

The findings of this study indicate that the direct effect of a growth mindset on student writing feedback literacy was not significant. Although the correlational analysis suggested a significantly positive association between the two variables (*r* = 0.364, *p* < 0.01), this direct path disappeared once achievement goals were introduced as mediators in the structural equation model. These results suggest that a growth mindset, as a distal motivational belief, influences student writing feedback literacy through achievement goals as proximal self-regulatory mechanisms (Elliot and Church, 1997; Burnette et al., 2013; Lee and Bong, 2019). Similarly, research on L2 writing feedback indicates that a growth mindset influences feedback-seeking behavior indirectly through achievement goals (Yao and Zhu, 2024). Although their study focused on feedback-seeking behavior rather than student writing feedback literacy, both constructs reflect students’ active engagement in feedback processes. In contrast, Zhu et al. (2024) found that a growth mindset significantly and positively predicted all five dimensions of student writing feedback literacy in an L1 writing context. Such differences indicate that a growth mindset may influence student writing feedback literacy through different pathways in L1 and L2 writing contexts, which may be related to how cognitive resources are allocated during L2 writing tasks.

Writing is constrained by working-memory capacity (Kellogg, 1996). In contrast to the more automatized language processing in L1 writing, L2 writing requires sustained cognitive resources for lexical retrieval, grammatical encoding, and syntactic organization (Kormos, 2012; Li, 2023). Under such conditions, fewer cognitive resources remain available for revision and feedback processing (Schoonen et al., 2009; Li and Roshan, 2019). Given that student writing feedback literacy involves understanding feedback, regulating affective responses, and turning feedback into revision actions (Carless and Boud, 2018; Yu and Liu, 2021), its development in L2 writing is likely constrained by limited cognitive resources. In this context, a growth mindset, as a distal belief, may not directly shape how students process feedback during specific writing tasks. Achievement goals, as a proximal self-regulatory mechanism, more directly shape students’ understanding of feedback, regulation of affective responses, and transformation of feedback into revision actions. Consequently, in cognitively demanding L2 writing contexts, a growth mindset may not directly predict student writing feedback literacy; its influence may operate through achievement goals.

### 5.2 The mediating effects of achievement goals between growth mindset and student writing feedback literacy

This study found that achievement goals fully mediated the influence of a growth mindset on student writing feedback literacy. Growth mindset was positively associated with development-approach, development-avoidance, and demonstration-approach goals, all of which served as mediators in the relationship between growth mindset and student writing feedback literacy. Demonstration-avoidance goals were not associated with either growth mindset or student writing feedback literacy.

Development-approach goals served as a positive mediator in the relationship between growth mindset and student writing feedback literacy. This indicates that Japanese learners with a growth mindset are more likely to view language ability as improvable through effort and strategic learning, thereby fostering development-approach goals, which orient learners toward competence development, effective emotion regulation, sustained positive learning experiences, and persistent engagement in learning activities (Zeng et al., 2016; Lou and Noels, 2017, 2019; Ma et al., 2024). Previous research has confirmed that the combination of a growth mindset and development-approach goals promotes learners’ writing feedback-related behavioral responses, including writing self-regulation, feedback-seeking behaviors, and improved writing performance (Limpo and Alves, 2017; Camacho et al., 2022; Zhang and Jiang, 2024; Yao and Zhu, 2024). The present study further shows that this combination enables Japanese learners to view feedback as a resource for personal growth, to be open to feedback from multiple sources, to make accurate judgments, to regulate emotions effectively, and to take purposeful action to enhance their writing (Carless and Boud, 2018; Yu et al., 2022).

Development-avoidance goals also positively mediated the relationship between a growth mindset and student writing feedback literacy, although the effect was small in magnitude. Compared with previous studies that have typically characterized development-avoidance goals as reflecting defensive motivation and having limited influence on how L2 students handle feedback (Papi et al., 2019; Sun and Huang, 2023), the present findings suggest that their role may be somewhat more positive than previously assumed. Within the 2 × 2 achievement goal model, development-avoidance goals are viewed as a hybrid goal orientation because they simultaneously involve both adaptive (development-focused) and maladaptive (avoidance-focused) components (Elliot and Hulleman, 2017). The effects of such goals may therefore depend on which component becomes more salient in a given learning context (Korn and Elliot, 2016). Prior research suggests that development-avoidance goals tend to show weaker and less consistent adaptive effects than development-approach goals, but more adaptive consequences than demonstration-avoidance goals, a pattern that is consistent with the small positive mediating effect observed in the present study (Korn and Elliot, 2016; Elliot and Hulleman, 2017). The present study found an association between growth mindset and development-avoidance goals, consistent with previous research (Lou and Noels, 2017; Papi et al., 2019, 2021). Because a growth mindset emphasizes the belief that ability can be improved through sustained effort and strategic regulation, it may activate the development-oriented component of development-avoidance goals. This orientation may help students recognize the learning-related information and improvement potential embedded in feedback, leading them to interpret feedback as a resource for maintaining or promoting competence rather than as a threat to ability. Development-avoidance goals are more likely to emerge when individuals have acquired a certain level of competence but begin to perceive instability or risk of decline (Elliot and Hulleman, 2017), a condition that plausibly characterizes the advanced Japanese learners examined in the present study, whose writing development often lags behind other language skills and involves frequent exposure to errors. In such contexts, concerns about preventing decline may motivate more careful and deliberate engagement with feedback, including closer evaluation and strategic use of feedback to maintain or gradually enhance writing performance. Moreover, the process-oriented nature of L2 writing and feedback, which emphasizes iterative revision and ongoing improvement (Hyland and Hyland, 2019), may further strengthen the development-oriented component of development-avoidance goals.

Demonstration-approach goals mediated the relationship between growth mindset and student writing feedback literacy, reflecting a similar motivational pattern to that reported by Zhang and Jiang (2024) and Yao and Zhu (2024). This finding supports the view discussed earlier that students with a growth mindset may pursue demonstration-approach goals (Molden and Dweck, 2000; Yu and McLellan, 2020). In L2 writing feedback contexts, students with a growth mindset are more likely to construe demonstration-approach goals as opportunities to demonstrate their current writing competence and to interpret feedback as diagnostic information about their present writing ability, which may in turn contribute to higher levels of student writing feedback literacy. Papi et al. (2019) reported that learners with a growth mindset and demonstration-approach goals tend to perceive feedback as high in value and low in ego and self-presentation cost. Notably, among the four achievement goal types, demonstration-approach goals exhibited the strongest predictive power for student writing feedback literacy and generated the most substantial mediating effect. The following discussion proposes several contextual and cultural interpretive hypotheses to help account for this result within the specific instructional context. One possible explanation concerns the assessment practices used in Japanese writing courses at universities. In these courses, feedback is typically delivered through scores and grades, with limited process-oriented guidance. Such assessment practices make students more sensitive to grades and external standards, thereby heightening their attention to external evaluation. Another explanation concerns the considerable examination pressure experienced by Asian students, which contributes to a broader evaluation-focused value system (Dweck, 1999). For senior Japanese majors in particular, higher scores are closely tied to advantages in postgraduate admissions, employment opportunities, and qualification examinations; this competitive environment may strengthen the pathway from growth mindset to demonstration-approach goals. Finally, in East Asian collectivist contexts, social-oriented achievement motivation (SOAM) has been widely discussed as a salient factor, and students may pursue social approval by meeting externally defined standards (Tao et al., 2023). Such a cultural orientation may also provide favorable conditions for the positive role of demonstration-approach goals in feedback engagement. Overall, in contexts where grades and external evaluations are emphasized, students with a growth mindset are more likely to adopt demonstration-approach goals, thereby facilitating the development of student writing feedback literacy.

Demonstration-avoidance goals were found neither to mediate the relationship between growth mindset and student writing feedback literacy nor to show significant associations with either construct. Demonstration-avoidance goals stem from a heightened perception of failure as threatening and are characterized by a self-defensive motivational orientation centered on avoiding failure and negative social evaluation (Elliot and Harackiewicz, 1996; Korn and Elliot, 2016). In L2 learning contexts, such goals are typically associated with maladaptive outcomes, including weaker L2 willingness to communicate (Feng et al., 2023) and reduced learning engagement and achievement (Wang et al., 2023). In contrast, students with a growth mindset tend to view failure as temporary and informative feedback rather than as a negative evaluation of stable ability (Dweck and Leggett, 1988; Dweck, 2006). Because a growth mindset weakens the perceived threat of failure, students have little need to employ failure-avoidant strategies to protect self-worth. Previous research has shown that the association between growth mindset and demonstration-avoidance goals is typically weak or even negative (Robins and Pals, 2002; Burnette et al., 2013; Papi et al., 2019). Student writing feedback literacy further requires students to engage actively with feedback processes, including understanding, evaluating, and using feedback for improvement. However, for students with demonstration-avoidance goals, writing feedback is often interpreted as a signal of failure and incompetence, which activates defensive responses related to threats to self-esteem and social image and leads to avoidance of feedback (Lou and Noels, 2017, 2019). Accordingly, such students tend to assign low value to feedback (Papi et al., 2021), are unlikely to seek it actively (Sun and Huang, 2023), and often perceive the psychological costs of feedback as outweighing its potential learning benefits (Papi et al., 2019). Collectively, demonstration-avoidance goals are unlikely to function as a mediating pathway linking growth mindset to student writing feedback literacy.

## 6 Conclusion and implications

This study examined the relationships among growth mindset, achievement goals, and student writing feedback literacy among Japanese learners through a questionnaire-based survey. The results showed that growth mindset significantly and positively predicted development-approach, development-avoidance, and demonstration-approach goals, which in turn mediated the relationship between growth mindset and student writing feedback literacy. Notably, growth mindset did not directly predict student writing feedback literacy; instead, its influence operated primarily through the mediating effects of achievement goals. This indicates that, in L2 writing feedback contexts, students’ beliefs in the malleability of language ability contribute to the development of student writing feedback literacy only when such beliefs are translated into concrete goal orientations. In particular, the demonstration-approach goal emerged as the strongest mediator in the model. This finding not only corroborates prior research showing that demonstration-approach goals can be activated through a growth mindset to produce adaptive outcomes, such as feedback-seeking behaviors and self-regulatory strategies, but also extends this line of work by demonstrating the particularly prominent position of demonstration-approach goals in L2 writing feedback as a prototypical evaluative learning activity. In this context, learners tend to view feedback as an opportunity to show their improvement and to use comments to revise and improve their writing. Moreover, the present study indicates that demonstration-approach goals contribute not only to feedback-related behaviors but also to the development of student writing feedback literacy, which is a multidimensional construct encompassing students’ cognitive understanding of feedback, affective engagement with evaluation, and behavioral responses in writing. Overall, the findings underscore the pivotal bridging role of achievement goals in connecting growth mindset with student writing feedback literacy, thereby providing new evidence for understanding how individual learner factors shape the development of feedback literacy in L2 writing contexts.

Building on these findings, several pedagogical implications can be drawn for L2 writing instruction. With respect to instructional goals, teachers should foreground a development-oriented approach that emphasizes the malleability of language ability and encourages continual improvement rather than focusing solely on grades. As prior research has shown, teachers’ feedback practices can implicitly convey beliefs about whether language ability is fixed or improvable, thereby shaping students’ mindsets, perceived support, and motivation (Lou and Noels, 2019). Feedback that attributes success or difficulty to effort, strategy use, and learning processes, rather than fixed ability, is more likely to foster growth-oriented beliefs and adaptive motivational responses. At the same time, although the present study finds an adaptive role of demonstration-approach goals in L2 writing feedback, previous research has cautioned that an excessive emphasis on performance display and outcome-based evaluation may heighten evaluative pressure and increase learners’ sensitivity to external judgment. This underscores the importance of carefully designed assessment practices (Korn and Elliot, 2016; Lou and Noels, 2019). In writing assessment, teachers should reduce their reliance on summative scores and make greater use of formative assessment practices, including multiple revisions, staged feedback, and progress-based evaluations. Such practices can help learners perceive feedback not as a final judgment of ability, but as a learning resource that provides concrete guidance for improvement, while also preventing the overactivation of demonstration-avoidance goals. Regarding feedback practices, integrating peer review, collaborative feedback activities, and structured self-reflection within a supportive classroom climate can encourage learners to actively seek, interpret, and use feedback. Such practices can gradually enhance their student writing feedback literacy.

## 7 Limitations and future research

As an initial exploratory study, several limitations should be acknowledged. First, the participants were drawn from a single university, which limits the representativeness of the sample. Future research may broaden the sample size and include learners from different regions and educational levels to enhance the generalizability of the findings. Second, despite its acceptable reliability, the use of a shortened three-item measure of growth mindset may limit the content validity of the construct, as it may not fully capture all facets of L2 mindsets. Future studies are therefore encouraged to employ the full L2B scale or incorporate scales measuring age sensitivity beliefs about language learning to provide a more comprehensive assessment of learners’ language mindsets. Third, the cross-sectional design constrains a full understanding of causal relationships and dynamic developmental patterns among the variables, and future research could incorporate longitudinal designs or multi-group comparisons to further examine the model’s stability and applicability across different contexts.

## Data Availability

The original contributions presented in the study are included in the article/supplementary material, further inquiries can be directed to the corresponding author.

## Footnotes

1
https://www.wjx.cn/
